# Brazilian Black Women are at Higher Risk for COVID-19 Complications: An Analysis of REBRACO, a National Cohort

**DOI:** 10.1055/s-0043-1770133

**Published:** 2023-06-20

**Authors:** Amanda Dantas-Silva, Fernanda Garanhani Surita, Renato Souza, Leila Rocha, José Paulo Guida, Rodolfo Pacagnella, Ricardo Tedesco, Karayna Fernandes, Sérgio Martins-Costa, Frederico Peret, Francisco Feitosa, Evelyn Traina, Edson Cunha Filho, Janete Vettorazzi, Samira Haddad, Carla Andreucci, Mario Correa Junior, Marcos Dias, Leandro de Oliveira, Elias Melo Junior, Marília Luz, Jose Guilherme Cecatti, Maria Laura Costa

**Affiliations:** 1 Universidade Estadual de Campinas, Campinas, SP, Brazil; 2 Jundiaí School of Medicine, Jundiaí, SP, Brazil; 3 Clinics Hospital of Porto Alegre, Porto Alegre, RS, Brazil; 4 UNIMED Maternity, Belo Horizonte, MG, Brazil; 5 Universidade Federal do Ceará, Fortaleza, CE, Brazil; 6 Escola Paulista de Medicina, Universidade Federal de São Paulo, São Paulo, SP, Brazil; 7 Moinhos de Vento Hospital, Porto Alegre, RS, Brazil; 8 Jorge Rossmann Regional Hospital – Sócrates Guanaes Institute, Itanhaém, SP, Brazil; 9 Universidade Federal de São Carlos, São Carlos, SP, Brazil; 10 Sumaré State Hospital, Sumaré, SP, Brazil; 11 Universidade Federal de Minas Gerais, Belo Horizonte, MG, Brazil; 12 Fernandes Figueira Institute, Rio de Janeiro, RJ, Brazil; 13 Botucatu Sao Paulo State University School of Medicine, Botucatu, SP, Brazil; 14 Universidade Federal de Pernambuco, Recife, PE, Brazil; 15 Santa Casa de Misericórdia of Pará, Belém, PA, Brazil

**Keywords:** COVID-19, Obstetrics, Racial disparities, Black women, COVID-19, Obstetrícia, Disparidades raciais, Mulheres negras

## Abstract

**Objective** To evaluate the impact of the race (Black versus non-Black) on maternal and perinatal outcomes of pregnant women with COVID-19 in Brazil.

**Methods** This is a subanalysis of REBRACO, a Brazilian multicenter cohort study designed to evaluate the impact of COVID-19 on pregnant women. From February 2020 until February 2021, 15 maternity hospitals in Brazil collected data on women with respiratory symptoms. We selected all women with a positive test for COVID-19; then, we divided them into two groups: Black and non-Black women. Finally, we compared, between groups, sociodemographic, maternal, and perinatal outcomes. We obtained the frequency of events in each group and compared them using X2 test; p-values < 0.05 were considered significant. We also estimated the odds ratio (OR) and confidence intervals (CI).

**Results** 729 symptomatic women were included in the study; of those, 285 were positive for COVID-19, 120 (42.1%) were Black, and 165 (57.9%) were non-Black. Black women had worse education (p = 0.037). The timing of access to the health system was similar between both groups, with 26.3% being included with seven or more days of symptoms. Severe acute respiratory syndrome (OR 2.22 CI 1.17–4.21), intensive care unit admission (OR 2.00 CI 1.07–3.74), and desaturation at admission (OR 3.72 CI 1.41–9.84) were more likely to occur among Black women. Maternal death was higher among Black women (7.8% vs. 2.6%, p = 0.048). Perinatal outcomes were similar between both groups.

**Conclusion** Brazilian Black women were more likely to die due to the consequences of COVID-19.

## Resumo

**Objetivo** Avaliar o impacto da raça (negra versus não negra) nos desfechos maternos e perinatais de gestantes com COVID-19 no Brasil.

**Métodos** Esta é uma subanálise da REBRACO, um estudo de coorte multicêntrico brasileiro desenhado para avaliar o impacto da COVID-19 em mulheres grávidas. De fevereiro de 2020 a fevereiro de 2021, 15 maternidades do Brasil coletaram dados de mulheres com sintomas respiratórios. Selecionamos todas as mulheres com teste positivo para COVID-19; em seguida, as dividimos em dois grupos: mulheres negras e não negras. Finalmente, comparamos, entre os grupos, os resultados sociodemográficos, maternos e perinatais. Obtivemos a frequência dos eventos em cada grupo e comparamos usando o teste X2; Valores de p < 0,05 foram considerados significativos. Também estimamos o odds ratio (OR) e os intervalos de confiança (IC).

**Resultados** 729 mulheres sintomáticas foram incluídas no estudo; desses, 285 foram positivos para COVID-19, 120 (42,1%) eram negros e 165 (57,9%) não eram negros. As mulheres negras apresentaram pior escolaridade (p = 0,037). O tempo de acesso ao sistema de saúde foi semelhante entre os dois grupos, com 26,3% incluídos com sete ou mais dias de sintomas. Síndrome respiratória aguda grave (OR 2,22 CI 1,17–4,21), admissão em unidade de terapia intensiva (OR 2,00 CI 1,07–3,74) e dessaturação na admissão (OR 3,72 CI 1,41–9,84) foram mais prováveis de ocorrer entre mulheres negras. A mortalidade materna foi maior entre as negras (7,8% vs. 2,6%, p = 0,048). Os resultados perinatais foram semelhantes entre os dois grupos.

**Conclusão** Mulheres negras brasileiras tiveram maior probabilidade de morrer devido às consequências da COVID-19.

## Introduction

During pregnancy, COVID-19 has been associated with worse maternal and perinatal outcomes, such as a higher likelihood of admission to the Intensive Care Unit (ICU), requiring invasive ventilation, increased risk of preterm birth, pre-eclampsia, indication for C-sections, more significant admission to the neonatal ICU, and maternal death. 1

In Brazil, one of the countries that have arguably suffered the most from the pandemic, disparities according to skin color have also affected maternal mortality - with maternal deaths being twice as frequent in Black women compared to White women. 2 The consequences of the pandemic have thus exposed underlying healthcare delays and highlighted the vulnerability of the system's diverse and multi-racial population. 3

Brazil is known for its racial plurality but is also marked by structural and cultural racism. 4 According to the Brazilian Institute of Geography and Statistics (IBGE), 5 the Brazilian population is primarily Black (56.3%). Nevertheless, racism and racial disparities are perpetuated. It is known that structural racism is central to determining population health and there is increasing evidence of ethnic and racial disparities pervading health issues. 6 Concerning maternal health, Black women have the highest mortality and severe maternal morbidity rates in addition to delayed (or lack of) prenatal care, inappropriate health assistance, and worse experiences during pregnancy, childbirth, and postpartum. 7 8

The Brazilian network of COVID-19 during pregnancy initiative (REBRACO) is a multicenter cohort study aimed at evaluating the clinical and epidemiological characteristics of SARS-CoV-2 infection and its associated outcomes during pregnancy and postpartum in Brazil. 9 This analysis aimed to understand the impact of race on maternal and perinatal outcomes of Brazilian women with COVID-19.

## Methods

This is a secondary analysis of REBRACO (Brazilian Network of COVID-19 and Obstetrics, in the Portuguese acronym). REBRACO was a multicenter prospective cohort conducted from February 2020 until February 2021 that included 15 Brazilian maternity hospitals.

Methodological aspects and main findings of REBRACO have previously been published elsewhere. 9 10 11 Briefly, during the data collection period, all women with suspected SARS-CoV-2 infection attended at any center of those participating in REBRACO were invited to participate in the study after signing informed consent. Suspected SARS-CoV2 infection was considered when women presented any of the following signs and symptoms: fever, cough, nasal congestion, runny nose, dyspnea, chest pain, chills, diarrhea, vomiting, nausea, wheezing, dizziness, fatigue, myalgia, arthralgia, headache, sore throat, hyposmia/anosmia, ageusia, desaturation/oxygen saturation <95%, loss of consciousness, confusion, seizure, cyanosis, rash, skin ulcer, difficulty in swallowing, dehydration, inappetence, intercostal retraction, pain abdominal pain, conjunctivitis, lymphadenopathy, contractions, reduced fetal movements, vaginal bleeding and inability to walk. Participants were tested for SARS-CoV-2 infection according to the local availability of testing.

For this analysis, we selected all women with a positive test for SARS-CoV-2 and for whom data regarding racial status was available. We considered the IBGE criteria for skin color classification for the racial status analysis. 12 The IBGE classifies the Brazilian population into five categories based on skin color by asking individuals to self-identify as either White, Black, "Pardo" (brown), Yellow (East Asian), or Indigenous. 12 In Brazil, ethnicity is particularly complex due to great miscegenation, and the term "Pardo" thus represents a diverse range of ethnic-mixed backgrounds. 12 The IBGE categorizes Black people in Brazil as all people who identify as Black and Pardo. So, in this study, the category "Black woman" referred to women who self-declared as Black or "Pardo". In contrast, the category "non-Black woman" corresponds to the other three IBGE skin color categories (i.e., White, Yellow, and Indigenous). 12

The following characteristics were evaluated in the current study: sociodemographic (age, education, marital status, pre-gestational BMI, region), obstetric characteristics (multiple pregnancy, parity, planned or unplanned pregnancy, pregnancy or postpartum period, type of prenatal insurance), and previous maternal comorbidities (alcohol use, asthma, chronic kidney disease, diabetes, HIV infection, hypertension, and smoking). For descriptive purposes, the North and Northeast Brazilian regions were grouped. This information was collected at enrolment.

After the clinical presentation of a suspected case of COVID-19, we followed the women until delivery if pregnant or until resolution of the COVID-19 suspected case if postpartum at admission. Data related to the suspicious symptomatic COVID-19 infection, characteristics of the management and resolution of the suspected infection, pregnancy, and maternal and perinatal outcomes were collected through a review of medical records, telephone interviews with the women, and in-person interviews.

Medical chart data were registered in the online RedCap® platform (an encrypted database where all the participating investigators could insert and update confidential patient information). Research collaborators had hierarchical and clustered access to the system; data was properly anonymized and personal, and contact information was kept confidential. The STROBE Statement (Strengthening the Reporting of Observational Studies in Epidemiology) was followed. 13

For statistical analysis, women were divided into Black and Non-Black women. For bivariate analysis, we performed Chi-square or Fisher's exact tests (according to the number of subjects). A p-value < 0.05 was considered statistically significant. We also obtained the Odds ratio (OR), and respective 95% confidence intervals (CI) were calculated for conditions relating to care provision and outcomes according to skin color. We performed statistical analysis with the software EpiInfo 7.2.5.0 (Center for Disease Control, Atlanta, 2011).

The REBRACO study followed the Declaration of Helsinki amended in Hong Kong in 1964, and it was approved by the Institutional Review Board (IRB) of the coordinating center and by each participating center (Research Ethics Committee of the School Medical Science, Letters of Approval numbers 4.047.168, 4.179.679, and 4.083.988). All women invited to participate received detailed information about the study, the follow-up, and the data and sample collections, when applicable. Participating women signed written informed consent documents before being enrolled. Regarding the underage patients, written informed consent was obtained from their guardians before enrollment and after receiving complete information about the study.

## Results

**Fig. 1:**
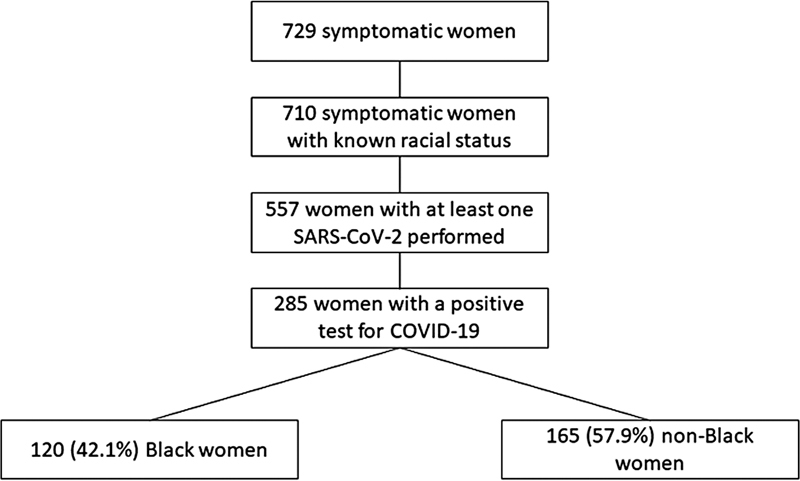
Flowchart of women included in this analysis.

A total of 729 women were included in the REBRACO cohort; of those, data regarding racial status was available for 710 women (301 (42.4%) Black women and 409 (57.6%) non-Black women). Of those, 557 underwent COVID-19 testing, according to each center's protocol. Two hundred eighty-five (285) women were positive for SARS-CoV-2 infection and were included in this analysis. Among those women, 120 (42.1%) were Black, and 165 (57.9%) were non-Black. Figure 1 presents the inclusion flowchart for this analysis.

Table 1 presents the sociodemographic and obstetrical characteristics of included women in this analysis. The majority of women included in both groups came from the Southeast region of Brazil, which is the largest in the country. Among Black women, the second most important region was North/Northeast. In contrast, in the other group, the second most important region was the South, expressing a national difference in racial distribution of the population (p-value <0.01). Black women had low educational levels (27.4% vs. 41.3%, p-value = 0.037). Another critical difference among both groups was regarding the source of payment for antenatal care: exclusive public funding occurred for 75.5% of Black women and 63.8% of non-Black women (p-value = 0.044).

**Table 1:** Sociodemographic and obstetrical characteristics of COVID-19 symptomatic women classified according to skin colour

Variable	Black	Non-Black	P-value
N	120 (42.1)	165 (57.9)	
Region			<0.01
North/Northeast	39 (32.5)	4 (2.4)	
Southeast	75 (62.5)	108 (65.5)	
South	6 (5.0)	53 (32.1)	
Marital Status			0.58
With partner	75 (64.1)	109 (67.3)	
Without partner	42 (35.9)	53 (32.7)	
Schooling			0.037
Secondary or less	74 (73.3)	82 (60.3)	
College or more	27 (26.7)	54 (39.7)	
Obesity	20 (27.4)	52 (41.3)	0.049
Health insurance of antenatal care			0.044
Public	80 (75.5)	102 (63.8)	
Private	26 (24.5)	58 (36.2)	
Parity			0.027
First pregnancy	50 (42.4)	49 (29.7)	
Two or more	68 (57.6)	116 (70.3)	
Unplanned pregnancy	46 (46.0)	94 (63.5)	>0.01
Pre-existing hypertension	9 (7.5)	16 (9.7)	0.517
Pre-existing diabetes	3 (2.5)	3 (1.8)	0.692
Asthma	12 (10.0)	9 (5.5)	0.147
Chronic kidney disease	1 (0.8)	0 (0)	0.240
Smoking	2 (1.7)	0 (0)	0.09

Table 2 shows some delays associated with care among women with COVID-19. Inclusion in the study after seven or more days of symptoms was considered a proxy for the delay to start care, and 29 (25.7%) Black women and 46 (29.7%) non-Black women had delays according to this criterion. Also, 17 (14.8%) and 22 (13.8%) women in the groups expressed difficulty in self-perception of illness; however, few reported difficulties accessing health services. Rates of delays were similar among both groups.

**Table 2:** Risk estimates for delays associated with care among women with COVID-19, according to race background

	Black	Non-Black	Odds ratio (Confidence interval)	p-value
Multiple testing	23 (19.3)	32 (19.6)	0.981 (0.540–1.782)	0.949
≥ 7 days with symptoms at enrolment	29 (25.7)	46 (29.7)	0.818 (0.474–1.411)	0.470
Difficulty in self-perception of illness	17 (14.8)	22 (13.8)	1.088 (0.549–2.156)	0.809
Difficulty in health services access	2 (1.8)	4 (2.5)	0.688 (0.124–3.819)	0.667

Black women had a higher frequency of adverse maternal outcomes, according to the results presented in Table 3 . Black women were more likely to be admitted with desaturation (OR 3.723, CI 1.408–9.844) and severe acute respiratory syndrome (OR 2.216, CI 1.166–4.211). The association of these conditions increased the intensive care unit admission among those women (OR 1.998, CI 1.067–3.743). Occurrence of maternal death was significantly higher among Black women: 9 (7.8%) deaths in this group, compared to 4 (2.6%) (p-value 0.048) in the other group.

**Table 3:** Risk estimates for adverse maternal outcomes in COVID-19 positive women classified according to skin colour

	Black	Non-Black	Odds ratio (Confidence interval)	p-value
SARS	27 (22.5)	19 (11.6)	2.216 (1.166–4.211)	0.014
ICU admission	27 (22.7)	21 (12.8)	1.998 (1.067–3.743)	0.029
Intubation	9 (8.1)	9 (6.7)	1.225 (0.469–3.201)	0.678
Pronation	9 (8.2)	5 (3.7)	2.299 (0.747–7.07)	0.137
Maternal Death	9 (7.8)	4 (2.6)	3.175 (0.953–10.580)	0.048
Desaturation	16 (13.7)	6 (4.1)	3.723 (1.408–9.844)	<0.01

We observed a high frequency of preterm delivery in our sample (32.5% and 29.2%, Black and non-Black women, respectively); however, it was similar between both groups; it probably impacted the frequency of neonatal intensive care unit admission (28.4% and 26.1%). The majority of women included in this analysis underwent cesarean section. These data is presented in Table 4 .

**Table 4:** Risk estimates for gestational and perinatal outcomes in confirmed COVID-19 women classified according to skin colour

	Black	Non-Black	Odds ratio (Confidence interval)	p-value
Fetal Death	2 (2.5)	1 (0.9)	2.897 (0.258–32.510)	0.367
Preterm delivery	26 (32.5)	33 (29.2)	1.167 (0.628–2.168)	0.624
Preeclampsia	10 (12.8)	11 (9.7)	1.364 (0.549–3.387)	0.503
Cesarean section	50 (62.5)	76 (66.1)	0.855 (0.472–1.550)	0.606
Small for gestational age	20 (29.0)	22 (22.4)	1.410 (0.697–2.851)	0.338
Large for gestational age	7 (12.5)	12 (13.6)	0.905 (0.333–2.457)	0.844
5th-minute Apgar < 7	4 (5.3)	5 (4.5)	1.189 (0.309–4.578)	0.801
NICU admission	21 (28.4)	29 (26.1)	1.120 (0.579–2.166)	0.735
Neonatal death	4 (5.5)	3 (2.7)	2.068 (0.449–9.522)	0.342

## Discussion

Our study compared maternal and perinatal outcomes of women included in the REBRACO study, a Brazilian national cohort of women with COVID-19. Our results showed that, despite having similar sociodemographic characteristics, Black women were more likely to present SARS, desaturation, and need for ICU admission. The frequency of death among Black women was higher when compared to non-Black women.

The results obtained after analyzing the sociodemographic characteristics were not surprising as a previous study, using data from the Brazilian population, reported similar findings. In that 2017 Brazilian study, having included 23 532 postpartum national women from 266 hospitals, it could be seen that the North and Northeast regions were more concentrated with Black women and showed a higher proportion of adolescent pregnancies. 14 In addition, Black women presented with less education and higher public insurance than non-Black women. Similarly, a previous national population survey showed higher unplanned pregnancy rates and greater use of public health services among Black women than White women. 15 Such findings reinforce how racial disparities are still very present in our population and illustrate how these marked sociodemographic differences may influence access to health services and the quality of care provided, supporting racial inequities in health. 16

Studies carried out in other countries have also pointed to socioeconomic differences between different racial groups as determinants of health. A cross-sectional analysis of survey data (between 2015 and 2017) from 107 921 women in 40 North American states showed lower rates of insurance among all categories of racial-ethnic minority women when compared to White, non-Hispanic women. 17

In our study, there was no significant difference between the skin color groups regarding the performance of multiple tests, readmission rate, delay in identifying those who were ill, or difficulty in reaching the health service. Nevertheless, Black women presented with more significant desaturation at admission when compared to non-Black women. Another Brazilian study (including 669 maternal SARS-CoV-2 cases) reported that Black women were more likely to be admitted with low O2 saturation at admission. 18 This factor may also be associated with the greater severity of adverse maternal outcomes detected in our analysis since low oxygen saturation at admission is associated with a higher risk for severe disease. 19

Black people, with the highest rates of perceived discrimination are generally associated with poorer health outcomes and even worse maternal outcomes. 5 7 20 Individuals who have reported any perceived medical setting discriminations in a medical setting have a higher frequency of reporting poor quality of care (e.g., not being allowed to partake in decision-making or not having enough time with the physician). Another effect among individuals who feel discriminated against may be the consequent underutilization of health services. 20 Therefore, it is possible that Black women avoid seeking health assistance because of perceived discrimination and subsequently obtain more severe clinical features.

Previous studies have shown Black skin color as a risk factor for worse adverse maternal outcomes (including maternal death) in women with COVID-19 infection. 1 2 3 In a cross-sectional study including 12,566 pregnant and postpartum women, Black women with any comorbidity had a 2-fold mortality rate when infected with SARS-CoV-2 as opposed to White women. 2 Another observational study of COVID-19 patients (not limited to obstetrics) showed that hospital admitted Mixed skin color (Pardo) Brazilians had a 1·45 higher risk of mortality, while Black Brazilians had a 1·32 higher risk of death. 3

Maternal death was 3-fold higher among Black women in our study. Still, when pregnant women (compared to non-pregnant women) have an increased risk for severe illness associated with COVID-19, the non-White skin color potentially adds additional clinical risk. 1 2 Historically, higher rates of severe maternal morbidity and mortality could be seen among Black women (compared to non-Black women), indicating that racial disparities are present in maternal mortality. 6 The COVID-19 pandemic has also exacerbated these inequalities. 21 Data on maternal mortality in Brazil due to COVID-19 have highlighted the inadequate monitoring of obstetric complications. 22 According to a cross-sectional observational study of COVID-19 hospital mortality using data from the SIVEP-Gripe with not only obstetrics patients, *Pardo* Brazilians admitted to hospital had 1.45 higher risk of mortality and Black Brazilian 1.32 higher risk of death than White ones. 3 Data from the Brazilian Official Acute Respiratory Syndrome Surveillance System (ARDS-SS), including 9563 pregnant and postpartum women with acute respiratory distress syndrome (ARDS), showed that 3·8% died with a confirmed diagnosis of COVID-19. 23 In our study, there were 13 maternal deaths representing 4.7% of the confirmed SARS-CoV-2 infected patients.

We did not find any significant differences in our study regarding preterm births. This was contrasting to previous literature where, in a retrospective cohort study with 162 pregnant and SARS-CoV-2 infected women, the preterm delivery rate was higher among Black women. 24

COVID-19 does not seem to be a democratic disease and has further exposed the strong association between race, ethnicity, culture, socioeconomic status, and health outcomes. 25 For example, despite being a middle-income country where the majority of the population is Black, the structural racism of Brazil (rooted in historical oppression and embedded in dominant cultures and social institutions that, in turn, led to poorer socioeconomic conditions) disproportionally made them the most vulnerable to COVID-19. 3

While this study has limitations, such as not being representative of the whole country and some regions being underrepresented, it provides some insight into well-documented data regarding the referral maternity hospitals involved in the care of pregnant and postpartum women that have tested positive for COVID-19.

Black women were already disproportionally affected before the pandemic, with the reasons for health system disparity being the same, i.e., implicit bias and structural racism. 26 Healthcare professionals might fail to recognize the effect of implicit bias in their practices, and this failure can potentially affect how obstetricians/gynecologists counsel patients. 13 It is, therefore, essential to broaden the debate and raise awareness of this issue, allowing for identifying and confronting practices that potentially result in verified inequities. Individual implicit bias and the profound impact of structural racism must be acknowledged and accepted before real progress can be made in reducing racial disparities in maternal mortality. However, it remains difficult to talk about racial health disparities in a country marked by structural racism. By helping shed some light on the health system-related discrimination and detrimental effects of SARS-CoV-2 on the Black population, this study hopes to expand the debate on racism in Brazil.

## Conclusion

Brazilian pregnant or postpartum Black women with COVID-19 were more likely to present desaturation, SARS, and ICU admission; maternal deaths were significantly higher among them compared to non-Black women. Urgent measures are needed to reduce racial disparities in pregnancy outcomes and discuss the causes of these disparities.

## References

[1] for PregCOV-19 Living Systematic Review Consortium Allotey J Stallings E Bonet M Clinical manifestations, risk factors, and maternal and perinatal outcomes of coronavirus disease 2019 in pregnancy: living systematic review and meta-analysis BMJ 2020 370 m3320 10.1136/bmj.m3320 32873575 PMC7459193

[2] Scheler C A Discacciati M G Vale D B Lajos G J Surita F Teixeira J C Mortality in pregnancy and the postpartum period in women with severe acute respiratory distress syndrome related to COVID-19 in Brazil, 2020 Int J Gynaecol Obstet 2021 155 03 475 482 10.1002/ijgo.13804 34185314 PMC9087770

[3] Baqui P Bica I Marra V Ercole A van der Schaar M Ethnic and regional variations in hospital mortality from COVID-19 in Brazil: a cross-sectional observational study Lancet Glob Health 2020 8 08 e1018 e1026 10.1016/S2214-109X(20)30285-0 32622400 PMC7332269

[4] Williamson K E The iatrogenesis of obstetric racism in Brazil: beyond the body, beyond the clinic Anthropol Med 2021 28 02 172 187 10.1080/13648470.2021.1932416 34180281

[5] Instituto Brasileiro de Geografia e Estatística Sistema IBGE de Recuperação Automática – SIDRA. Pesquisa Nacional por Amostra de Domicílios Contínua Anual [Internet]. 2021 [cited 2022 Nov 21]. Available from:https://sidra.ibge.gov.br/tabela/6408

[6] Bailey Z D Krieger N Agénor M Graves J Linos N Bassett M T Structural racism and health inequities in the USA: evidence and interventions Lancet 2017 389(10077):1453 1463 10.1016/S0140-6736(17)30569-X 28402827

[7] Chen J Cox S Kuklina E V Ferre C Barfield W Li R Assessment of incidence and factors associated with severe maternal morbidity after delivery discharge among women in the US JAMA Netw Open 2021 4 02 e2036148 10.1001/jamanetworkopen.2020.36148 33528553 PMC7856547

[8] Gadson A Akpovi E Mehta P K Exploring the social determinants of racial/ethnic disparities in prenatal care utilization and maternal outcome Semin Perinatol 2017 41 05 308 317 10.1053/j.semperi.2017.04.008 28625554

[9] REBRACO Study Group Costa M L Souza R T Pacagnella R C Brazilian network of COVID-19 during pregnancy (REBRACO: a multicentre study protocol) BMJ Open 2021 11 12 e051284 10.1136/bmjopen-2021-051284 PMC8685531 34921076

[10] REBRACO Study Group Guida J P Cecatti J G Souza R T Preeclampsia among women with COVID-19 during pregnancy and its impact on maternal and perinatal outcomes: Results from a national multicenter study on COVID in Brazil, the REBRACO initiative Pregnancy Hypertens 2022 28 168 173 10.1016/j.preghy.2022.05.005 35568019 PMC9085347

[11] REBRACO Study Group Souza R T Cecatti J G Pacagnella R C The COVID-19 pandemic in Brazilian pregnant and postpartum women: results from the REBRACO prospective cohort study Sci Rep 2022 12 01 11758 10.1038/s41598-022-15647-z 35817818 PMC9272878

[12] Osório R G O sistema classificatório de "cor ou raça" do IBGE Brasília (DF) Instituto de Pesquisa Econômica Aplicada 2003

[13] STROBE Initiative von Elm E Altman D G Egger M Pocock S J Gøtzsche P C Vandenbroucke J P The Strengthening the Reporting of Observational Studies in Epidemiology (STROBE) statement: guidelines for reporting observational studies PLoS Med 2007 4 10 e296 10.1371/journal.pmed.0040296 17941714 PMC2020495

[14] Leal M DC Gama S GND Pereira A PE Pacheco V E Carmo C ND Santos R V The color of pain: racial iniquities in prenatal care and childbirth in Brazil Cad Saude Publica 2017 33(33, Suppl 1):e00078816 10.1590/0102-311X00078816 28746555

[15] Theophilo R L Rattner D Pereira E L Vulnerabilidade de mulheres negras na atenção ao pré-natal e ao parto no SUS: análise da pesquisa da Ouvidoria Ativa Ciênc Saúde Coletiva 2018 23 11 3505 16 10.1590/1413-812320182311.31552016 30427424

[16] Saluja B Bryant Z How implicit bias contributes to racial disparities in maternal morbidity and mortality in the United States J Womens Health (Larchmt) 2021 30 02 270 273 10.1089/jwh.2020.8874 33237843

[17] Daw J R Kolenic G E Dalton V K Racial and ethnic disparities in perinatal insurance coverage Obstet Gynecol 2020 135 04 917 924 10.1097/AOG.0000000000003728 32168215 PMC7098441

[18] de Souza Santos D de Oliveira Menezes M Andreucci C B Disproportionate impact of Coronavirus Disease 2019 (COVID-19) among pregnant and postpartum black women in Brazil through structural racism lens Clin Infect Dis 2021 72 11 2068 2069 10.1093/cid/ciaa1066 32719866 PMC7454418

[19] Nascimento I JBD Pinto L R Fernandes V A Clinical characteristics and outcomes among Brazilian patients with severe acute respiratory syndrome coronavirus 2 infection: an observational retrospective study Sao Paulo Med J 2020 138 06 490 497 10.1590/1516-3180.2020.00365.R1.08092020 33263706 PMC9685581

[20] Boccolini C S Boccolini P M Damacena G N Ferreira A P Szwarcwald C L Factors associated with perceived discrimination in health services of Brazil: results of the Brazilian National Health Survey, 2013 Ciênc Saúde Coletiva 2016 21 02 371 378 10.1590/1413-81232015212.19412015 26910145

[21] Gurzenda S Castro M C COVID-19 poses alarming pregnancy and postpartum mortality risk in Brazil EClinicalMedicine 2021 36 100917 10.1016/j.eclinm.2021.100917 34124636 PMC8173266

[22] Nakamura-Pereira M Amorim M MR Pacagnella R C COVID-19 and maternal death in Brazil: an invisible tragedy Rev Bras Ginecol Obstet 2020 42 08 445 447 10.1055/s-0040-1715138 32898910 PMC10309250

[23] Nakamura-Pereira M Knobel R Menezes M O Andreucci C B Takemoto M LS The impact of the COVID-19 pandemic on maternal mortality in Brazil: 523 maternal deaths by acute respiratory distress syndrome potentially associated with SARS-CoV-2 Int J Gynaecol Obstet 2021 153 02 360 362 10.1002/ijgo.13643 33570755 PMC9087565

[24] Pope R Ganesh P Miracle J Structural racism and risk of SARS-CoV-2 in pregnancy EClinicalMedicine 2021 37 100950 10.1016/j.eclinm.2021.100950 34386742 PMC8343238

[25] Yaya S Yeboah H Charles C H Otu A Labonte R Ethnic and racial disparities in COVID-19-related deaths: counting the trees, hiding the forest BMJ Glob Health 2020 5 06 e002913 10.1136/bmjgh-2020-002913 PMC7298686 32513864

[26] Gillispie-Bell V The contrast of color: why the black community continues to suffer health disparities Obstet Gynecol 2021 137 02 220 224 10.1097/AOG.0000000000004226 33416278

